# Progranulin and diabetic kidney disease

**DOI:** 10.1186/1758-5996-7-S1-A13

**Published:** 2015-11-11

**Authors:** Bruna Bellincanta Nicoletto, Thaiana Cirino Krolikowski, Luis Henrique Santos Canani

**Affiliations:** 1 UFRGS, Porto Alegre, Brazil

## Background

Progranulin (PGRN) is expressed in many cell types, including adipocytes. It has been recognized as an adipokine related to obesity, insulin resistance and type 2 diabetes mellitus (T2D) and its levels depend on kidney function. However, the association of PGRN with diabetic kidney disease remains unknown.

## Objective

To evaluate serum and urinary levels of PGRN in patients with T2D and chronic kidney disease (CKD) stages 3-5 and compare to patients with T2D and glomerular filtration rate (GFR; CKD-EPI) >60 mL/min and with control individuals without T2D.

## Materials and methods

Case-control study. Cases were defined by the presence of T2D and CKD stages 3-5, evaluated by estimated GFR<60 mL/min, and controls were formed by patients with T2D and GFR>60mL/min (diabetic control group); and by individuals without T2D (non-diabetic control group). PGRN was determined with enzyme-linked immunosorbent assay in blood and urine samples after overnight fasting. The study groups were compared by ANOVA with Tukey or Kruskal-Wallis with Dunn tests for continuous and χ2 test for categorical variables. The Spearman's correlation coefficient was used. This study was approved by the Ethics Committee of Hospital involved, and all subjects signed the informed consent.

## Results

114 patients were included (25 at case group; 67 at T2D control group and 22 at non-diabetic control group). There were no differences in age, gender, ethnicity and body mass index (BMI) between groups. PGRN serum levels were increased in patients with T2D and CKD stages 3-5, when compared to control groups (cases: 71.97±21.75 vs. T2D control group: 57.39±17.99 and non-diabetic control group: 50.41±12.17 ng/dL; p<0.001). On the other hand, urinary PGRN was decreased in cases compared to T2D control group [cases: 10.62 (6.28-14.62) vs. T2D control group: 16.58 (10.15-24.11); non-diabetic control group: 13.51 (8.28-23.67) ng/dL; p=0.014]. There was a positive correlation between serum PGRN and BMI (r=0.246; p=0.008), waist circumference (r=0.236; p=0.012); ultra-sensitive C reactive protein (r=0.372; p<0.001) and interleukin-6 (r=0.350; p<0.001) and a negative correlation with GFR (r=-0.242; p=0.010) in all patients. Urinary PGRN was positively associated to urinary albumin excretion (r=0.256; p=0.007).

## Conclusion

PGRN serum levels seems to be a marker of obesity and inflammatory state that is affected by decrease in GFR; while urinary PGRN could be a marker of diabetic kidney disease.

